# Boosting Energy Deprivation by Synchronous Interventions of Glycolysis and Oxidative Phosphorylation for Bioenergetic Therapy Synergetic with Chemodynamic/Photothermal Therapy

**DOI:** 10.1002/advs.202401738

**Published:** 2024-03-15

**Authors:** Xiangjun Wei, Renlu Han, Yun Gao, Pengxin Song, Zhen Guo, Yafei Hou, Jiancheng Yu, Keqi Tang

**Affiliations:** ^1^ Institute of Mass Spectrometry School of Materials Science & Chemical Engineering Ningbo University Ningbo 315211 China; ^2^ Zhejiang Engineering Research Center of Advanced Mass Spectrometry and Clinical Application Zhenhai Institute of Mass Spectrometry Ningbo 315211 China; ^3^ Department of Microelectronics Science and Engineering School of Physical Science and Technology Ningbo University Ningbo 315211 China; ^4^ Faculty of Electrical Engineering and Computer Science Ningbo University Ningbo 315211 China

**Keywords:** bioenergetic therapy, chemodynamic therapy, CuFe_2_O_4,_ glycolysis, photothermal therapy

## Abstract

Bioenergetic therapy is emerging as a promising therapeutic approach. However, its therapeutic effectiveness is restricted by metabolic plasticity, as tumor cells switch metabolic phenotypes between glycolysis and oxidative phosphorylation (OXPHOS) to compensate for energy. Herein, Metformin (MET) and BAY‐876 (BAY) co‐loaded CuFe_2_O_4_ (CF) nanoplatform (CFMB) is developed to boost energy deprivation by synchronous interventions of glycolysis and OXPHOS for bioenergetic therapy synergetic with chemodynamic/photothermal therapy (CDT/PTT). The MET can simultaneously restrain glycolysis and OXPHOS by inhibiting hexokinase 2 (HK2) activity and damaging mitochondrial function to deprive energy, respectively. Besides, BAY blocks glucose uptake by inhibiting glucose transporter 1 (GLUT1) expression, further potentiating the glycolysis repression and thus achieving much more depletion of tumorigenic energy sources. Interestingly, the upregulated antioxidant glutathione (GSH) in cancer cells triggers CFMB degradation to release Cu^+^/Fe^2+^ catalyzing tumor‐overexpressed H_2_O_2_ to hydroxyl radical (∙OH), both impairing OXPHOS and achieving GSH‐depletion amplified CDT. Furthermore, upon near‐infrared (NIR) light irradiation, CFMB has a photothermal conversion capacity to kill cancer cells for PTT and improve ∙OH production for enhanced CDT. In vivo experiments have manifested that CFMB remarkably suppressed tumor growth in mice without systemic toxicity. This study provides a new therapeutic modality paradigm to boost bioenergetic‐related therapies.

## Graphical abstract

Metformin and BAY‐876 co‐loaded CuFe_2_O_4_ (CFMB) nanoplatform is developed to synchronously disturb glycolysis and oxidative phosphorylation for boosted bioenergetic therapy synergetic with chemodynamic/photothermal therapy. CFMB manifests potent in vivo tumor growth inhibition with good biosafety.

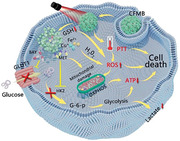

## 1 Introduction

Energy is the basis of cell life. To meet the highly bioenergetic demands, indefinitely proliferative tumor cells obtain large amounts of energy through glucose metabolism to produce adenosine triphosphate (ATP).^[^ ^1^ ^]^ Glycolysis, also known as the Warburg effect, is the predominant energy production pathway used by most tumors.^[^ ^2^ ^]^ It is reported that cancer cells have at least 30‐fold and tenfold enhancement in glycolysis rate and glucose consumption respectively in comparison with normal cells.^[^ ^3^ ^]^ Particularly, cancer cells produce more than 50 times more ATP through glycolysis than normal cells.^[^ ^4^ ^]^ This feature facilitates establishing creative approaches to targeted glycolysis for tumor treatment. In addition, oxidative phosphorylation (OXPHOS), another pathway for cancer cells to obtain energy, also proceeds at a high rate.^[^ ^5^ ^]^ The OXPHOS metabolic pathway generates ATP by the electron transport chain (ETC) of mitochondria.^[^ ^6^ ^]^ Because of metabolic heterogeneity and compensation, tumor cells switch metabolic phenotypes between glycolysis and oxidative OXPHOS to compensate for energy.^[^ ^7^ ^]^ In this context, simultaneously intervening glycolysis as well as OXPHOS via cooperation mechanisms are prospective means to improve antitumor bioenergetic therapy.

So far, many strategies have been developed to restrain glycolysis to reduce the energy supply of cancer cells, which can be generally categorized into two types: 1) inhibiting glycolytic key enzymes,^[^ ^8^ ^]^ 2) decreasing the intracellular glucose level.^[^ ^9^ ^]^ Targeting key enzymes at different stages of glycolysis is a promising approach. For example, suppression of hexokinase 2 (HK2) that catalyzes glucose to glucose‐6‐phosphate (G6P) in the first step of glycolysis would lead to failure of glucose phosphorylation,^[^ ^3^, ^8^ ^]^ decreased ATP production, and energy deprivation. Although promising, inhibition of enzyme activity alone does not achieve satisfactory energy‐deprivation effects due to intracellular glucose accumulation caused by blocked glucose metabolic pathways. The other strategy restraining glycolysis can be recognized as glucose starvation. A widely used scavenger for glucose is glucose oxidase (GOX) which can convert glucose into hydrogen peroxide (H_2_O_2_), which has gained significant attention owing to its outstanding anticancer ability via both inhibiting energy metabolism and killing cancer cells by H_2_O_2_.^[^ ^9a,b^ ^]^ Unfortunately, the GOX‐mediated glucose‐oxidation reaction heavily depends on oxygen (O_2_) concentration, thus the therapeutic efficiency is compromised by an insufficient O_2_ supply of tumors. In contrast, inhibiting glucose transporters to block glucose conveyance into tumor cells may be a better alternative. Glucose transporter 1 (GLUT1), a classic example of a glucose transporter, is universally overexpressed in cancer cells.^[^ ^9b^ ^]^ Targeting GLUT1 to block glucose transport has proven effective in both preclinical and clinical studies.^[^ ^9^, ^11^ ^]^ Therefore, dual‐pathway glycolysis inhibition by blocking upstream glucose supply while inhibiting downstream key enzyme activity might be a powerful and promising strategy for boosting energy deprivation of tumor cells.

As for the other OXPHOS energy acquisition pathway, the intervention strategy is mainly to induce mitochondrial function and structural damage,^[^ ^12^ ^]^ such as atovaquone (ATO) inhibiting mitochondrial respiration,^[^ ^13^ ^]^ Ca^2+^ inducing mitochondrial Ca^2+^‐overload,^[^ ^14^ ^]^ reactive oxygen species (ROS) reducing complex catalytic activity,^[^ ^15^ ^]^ H_2_S interrupting the ETC,^[^ ^16^ ^]^ and so on.^[^ ^17^ ^]^ OXPHOS inhibition has been demonstrated to be effective in reducing energy supply.^[^ ^18^ ^]^ Recently, poly(lactic‐co‐glycolic acid) co‐encapsulated pyropheophorbide and syrosingopine nanoparticles (mTPPa‐Sy NPs),^[^ ^19^ ^]^ aptamer‐based artificial enzyme,^[^ ^20^ ^]^ 3PO‐loaded cross‐linked sodium lipoic acid vesicle (3PO@cLANa),^[^ ^21^ ^]^ and so on^[^ ^20^, ^22^ ^]^ have been developed for targeting glycolysis and OXPHOS to regulate tumor metabolism, highlighting the effectiveness and necessity of synergetic energy deprivation. In these studies, the nano drugs interfere with glycolysis and OXPHOS through the single‐pathway action of related inhibitors and thus kill cancer cells only depending on the energy block. According to the concept of synthetic lethality,^[^ ^23^ ^]^ the single‐pathway suppression in cancer metabolism makes the other pathway essential for cell survival.^[^ ^19^ ^]^ In addition, given the heterogeneity and complexity of the tumor,^[^ ^24^ ^]^ energy deprivation alone might not be sufficient to eradicate the tumor. Chemodynamic therapy (CDT) catalyzes the decomposition of tumor‐overexpressed H_2_O_2_ into hydroxyl radicals (∙OH), which can not only impair OXPHOS but also kill cancer cells directly.^[^ ^25^ ^]^ More importantly, photothermal therapy (PTT) benefits in promoting CDT catalytic reaction.^[^ ^26^ ^]^ Based on these concerns, we hypothesized that synchronous interventions of glycolysis and OXPHOS through dual pathways, as well as introduction of CDT or/and PTT, would not only be lethal to energy deprivation but also potentiate tumor eradication.

**Scheme 1:**
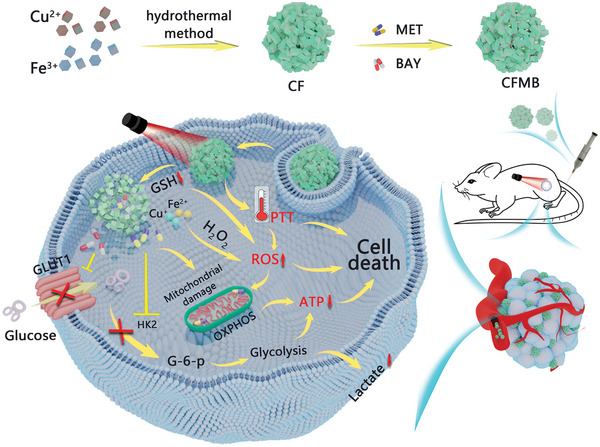
Scheme illustrating the synthesis of CFMB and its therapeutic mechanism for boosting energy deprivation by synchronous interventions of glycolysis and OXPHOS for bioenergetic therapy synergetic with CDT/PTT.

Herein, a versatile nano platform CuFe_2_O_4_@MET@BAY (CFMB) based on metformin (MET) and BAY‐876 (BAY) co‐loaded CuFe_2_O_4_ (CF) was constructed to boost energy deprivation by synchronous interventions of glycolysis and OXPHOS for antitumor bioenergetic/CDT/PTT therapy. **Scheme** **1** illustrates that CFMB possesses multiple merits: 1) CFMB can efficiently deliver drugs to the tumor area, where it is subsequently triggered by tumor‐overexpressed antioxidant glutathione (GSH) to degrade and release MET and BAY. 2) MET could synchronously suppress glycolysis and OXPHOS by inhibiting HK2 activity and damaging mitochondrial function to deprive energy, respectively. 3) BAY blocks glucose uptake by effectively inhibiting GLUT1 expression, further potentiating the glycolysis repression and thus achieving much more depletion of tumorigenic energy sources. 4) GSH‐triggered CFMB degradation releases Fenton‐like/Fenton reagent Cu^+^/Fe^2+^ ions to catalyze tumor‐overexpressed H_2_O_2_ to ∙OH, both impairing OXPHOS and achieving GSH‐depletion amplified CDT. (5) Upon 808 nm light irradiation, CFMB has a photothermal conversion capacity to kill cancer cells for PTT and improve ∙OH production for enhanced CDT. By combining with the above characteristics, CFMB achieves potent antitumor efficacies by boosting bioenergetic therapy and CDT/PTT in a mutually reinforcing way, providing a novel approach to designing bioenergetic‐based synergistic cancer treatment.

## 2 Results and Discussion

### 2.1 Synthesis and Characterization of CFMB NPs

**Figure 1:**
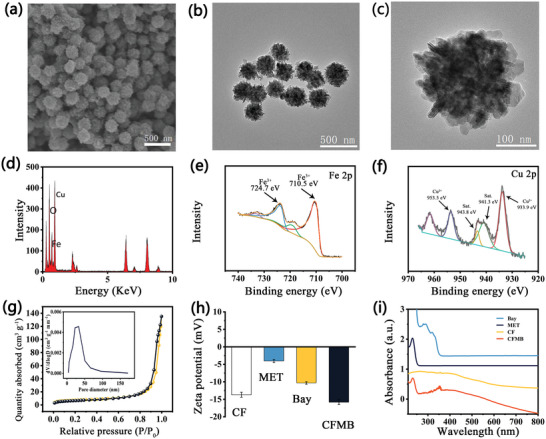
a) SEM image CF NPs. b,c) TEM images of CF NPs. d) EDS of CF NPs. High‐resolution e) Cu 2p, and f) Fe 2p XPS spectra. g) N_2_ adsorption–desorption isotherm of CF. (Inset: pore‐size distribution of CF.) h) Zeta potentials, and i) UV–vis spectra of CF, MET, BAY, and CFMB.

As shown in Scheme 1, CF NPs were successfully prepared using the one‐pot solvothermal method. The scanning electron microscopy (SEM) image displays that the as‐prepared CF NPs exhibited 3D flower‐like spherical structures (**Figure** **1**a). The detailed size distribution based on SEM images shows that CF NPs have an average diameter of 200 nm (Figure S1, Supporting Information). As can be seen from the transmission electron microscopy (TEM) image (Figure 1b), CF NPs show monodispersity and no agglomeration could be observed. A TEM image of individual CF nanoparticles in Figure 1c more clearly reveals the flower‐like CF was assembled from numerous nanosheets, which would provide lots of chemical reaction sites for catalytic reactions. X‐ray diffraction (XRD) analyses indicate the characteristic peaks in as‐prepared CF NPs could be assigned to the standard pattern (JCPDS NO. 25–0283) of CuFe_2_O_4_ (Figure S2, Supporting Information). The high‐resolution TEM (HRTEM) of CF displays distinct lattice fringes of 0.2956 nm (Figure S3, Supporting Information), which agrees with the lattice spacing of (220) plane of CuFe_2_O_4_, proving the spinel structure. Energy‐dispersive X‐ray spectroscopy (EDS) (Figure 1d) of CF demonstrates the existence of Cu, Fe, and O in these NPs and reveals the composition (Figure S4, Supporting Information). The high‐resolution X‐ray photoelectron spectroscopy (XPS) analysis of Fe 2p displayed the characteristic peaks at 710.5 and 724.7 eV belonging to Fe^3+^, demonstrating that Fe^3+^ is the only oxidation state present in CF NPs (Figure 1e). In the Cu 2p XPS spectrum, two signal peaks appeared at 933.9 and 953.5 eV with the satellite signals at 941.3 and 943.8 eV could be ascribed to Cu^2+^ (Figure 1f). The porosity of the CF was studied by N_2_ adsorption isotherm analysis as shown in Figure 1g. The adsorption hysteresis phenomenon demonstrates the porous structure of CF, and its BET surface area and pore size were determined to be 26.7 m^2^ g^−1^ and 26 nm respectively, which is more conducive to efficient drug loading. With the advantages of mesoporous structure, MET and BAY drug molecules were loaded and stored in the pores of CF by a simple mixing method. The Zeta potential decreases from −13.7 to −15.8 mV after BAY and MET are loaded due to their negative potentials (Figure 1h). The new absorption band at 230 nm corresponds to the characteristic peaks of MET, and the absorption at 350 nm is attributed to the characteristic peak redshift of BAY (Figure 1i). Although the absorption peak of MET in the UV region overlaps with that of BAY, the loading content of MET and BAY calculated by standard calibration curves is 4.13% and 1.63% respectively (Figure S5, Supporting Information), further confirming that MET has been successfully loaded in CF NPs. The element mapping of CFMB illustrates the homogeneous distribution of Cu, Fe, and F elements among the CFMB framework (Figure S6, Supporting Information), further proving the successful loading of BAY. The stability of the CFMB in H_2_O, phosphate buffer solutions (PBS), or Dulbecco's modified eagle medium (DMEM) was investigated by dynamic light scattering (DLS) (Figure S7, Supporting Information), where CFMB shows negligible hydrodynamic diameter change upon 7 days, proving its high stability.

### 2.2 Photothermal Performance

**Figure 2:**
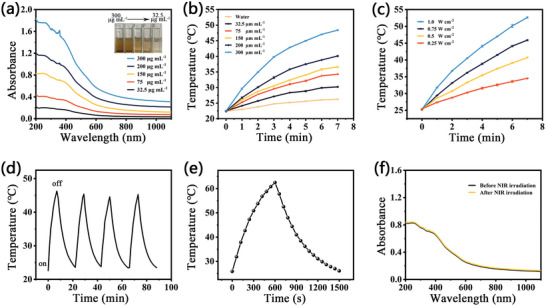
a) UV–vis–NIR absorption spectra of CF aqueous dispersion at various concentrations. b,c) Temperature curves of CF aqueous dispersion exposure to 808 nm light. d) The temperature curve of CF suffered four light on/off cycles. e) Heating‐cooling curves of CF under 808 nm irradiation as well as photothermal conversion efficiency. f) UV–vis–NIR absorbance spectra of CF before and after 808 nm irradiation.

As displayed in **Figure** **2**a, CF NPs have a wide absorption band in the near‐infrared (NIR) region, which is concentration‐dependent. The absorbance value in Figure S8 (Supporting Information) increases linearly as NP concentration increases. Meanwhile, the color of CF solutions gradually deepens, and all show good dispersion and light transmission (Figure 2a, inset). After irradiation with 808 nm light for 7 min, the temperature of CF NP aqueous dispersion increased by 7.8, 11.7, 14, 17.5, and 22.5 °C at concentrations of 32.5, 75, 150, 200, and 300 µg mL^−1^, respectively (Figure 2b). By contrast, the temperature of pure water rises by only 3.5 °C under the same condition. The above result suggests that CF could be a potential PTT reagent with excellent photothermal conversion ability. Moreover, its photothermal performance is laser power density‐dependent (Figure 2c). After four heating/cooling cycles for CF NPs, the temperature change remains stable (Figure 2d), demonstrating the excellent photostability of CF NPs. Its photothermal conversion efficiency (η) is as high as 35.2% (Figure 2e; Figure S9, Supporting Information). The negligible change could be found in its UV–vis–NIR absorbance spectrum after laser irradiation, further demonstrating the photostability of CF NPs (Figure 2f). The excellent η value and remarkable photostability make CF a promising PTT reagent.

### 2.3 GSH‐Triggered Drugs Release and Catalytic Reactions

**Figure 3:**
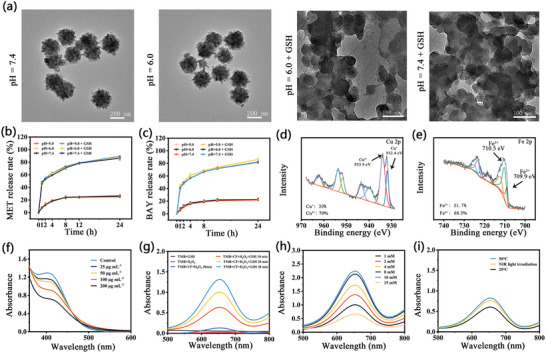
a) TEM images of CFMB NPs subjected to different treatments for 24 h. The release profile of b) MET and c) BAY at various conditions. High‐resolution d) Cu 2p, and e) Fe 2p XPS spectra of CF NPs treated by GSH. f) UV–vis spectra of DTNB treated with different concentrations of GSH in the presence of CF. g) UV–vis spectra of TMB after different treatments. h) UV–vis spectra of TMB treated by diverse GSH concentrations. i) UV–vis spectra of TMB at 25, 48 °C, and NIR light irradiation.

As shown in Scheme 1, the intervention of tumor metabolism is initiated by GSH‐mediated degradation of CFMB NPs, and the structure collapsing and degradation of CFMB NPs would lead to the release of loaded MET and BAY, as well as a series of catalytic reactions. The biodegradation behavior of CFMB in different environments is directly demonstrated by TEM images. Phosphate buffer solutions (PBS) were used to simulate complex physiological environments. When immersed in PBS with pH 7.4 or 6.0, the structure of CFMB NPs remained intact for 24 h (**Figure** **3**a), suggesting CFMB could keep the integrity of its original structure during blood circulation or at weakly acidic conditions. However, in the presence of GSH, the structure of CFMB completely collapsed at both pH 7.4 and 6.0 conditions, indicating GSH‐triggered degradation. The degradation of CFMB would facilitate the loaded BAY and MET release. Thus, drug release properties are subsequently studied. As shown in Figure 3b, ≈25% of MET was released at pH 7.4, 6.0, and 5.0 conditions, and once GSH was added, the drug release increased rapidly, and ≈90% MET was released within 24 h. Similarly, the cumulative BAY release in the presence of GSH is ≈4 times higher than that without GSH (Figure 3c). The release of MET and BAY at pH 6.0 condition increased with the elevation of GSH concentration (Figure S10, Supporting Information). The above result proves that CFMB possesses excellent GSH‐triggered degradation performance for subsequent drug release at the tumor site with GSH overexpression, which makes it a promising drug delivery system for biomedical applications. In addition, the CF NPs still retained a photothermal effect after descending into the ionic (Figure S11, Supporting Information).

Importantly, the XPS survey of CF NPs after reacting with GSH was conducted to further reveal the transformation of Cu^+^/Cu^2+^ and Fe^2+^/Fe^3+^ redox couple. High‐resolution Cu 2p XPS spectrum shows the coexistence of Cu^+^ and Cu^2+^ in CF NPs (Figure 3d), where the fitting peak intensity of Cu^2+^ at 933.9 eV decreases significantly, and the characteristic peak at 932.4 eV assigned to the emerged Cu^+^. Similarly, the fitting peaks of 709.9 eV attribute to the emerged Fe^2+^ in the Fe 2p XPS spectrum, confirming the success of the Fe^2+^/Fe^3+^redox couple conversion. According to XPS results, the content of Cu^+^ increases from 0% to 30%, while the proportion of Fe^2+^ increases to 31.7% after the reaction of CF with GSH. Furthermore, the consumption of GSH was assessed by 5,5′‐Dithiobis‐(2‐nitro benzoic acid) (DTNB). The product of DTNB reduced by GSH has a characteristic absorption peak at 412 nm.^[^ ^27^ ^]^ As displayed in Figure 3f, the absorption value at 412 nm declines dramatically as CF concentration increases, demonstrating the capability of CF to deplete GSH is concentration‐dependent. The above results prove that Cu^2+^ and Fe^3+^ in CF react with GSH to produce new Cu^+^ and Fe^2+^ ions, which may enable CF NPs to act as Fenton‐like/Fenton reagents to transform tumor‐overexpressed H_2_O_2_ into toxic ∙OH for CDT applications.

Subsequently, the ability of CF to produce ∙OH through a Fenton‐like/Fenton reaction was investigated by well‐developed 3,3,5,5‐tetramethylbenzidine (TMB) oxidation experiments in H_2_O_2_ dispersion of CF NPs with or without GSH. The product of TMB oxidized by ∙OH has a 652 nm absorption peak.^[^ ^15^ ^]^ The 652 nm peak shows a negligible change even after CF NPs have reacted with H_2_O_2_ for 30 min (Figure 3g), indicating no ∙OH produced. However, after adding GSH, the peak value at 652 nm continuously increases with the extension of time, suggesting the reaction of CF and GSH successfully occurs to generate Fenton‐like/Fenton reagent Cu^+^/Fe^2+^ ions which catalyzes H_2_O_2_ to ∙OH. At the fixed concentrations of CF and H_2_O_2_, the characteristic absorbance at 652 nm increases with increasing GSH concentration (1–10 mm, Figure 3h), while the GSH amount further increases (15 mm), the 652 nm absorbance decreases significantly, which could be attributed to the excess GSH in turn scavenging the generated ∙OH. In addition, the promoted ∙OH generations by the photothermal effect have been investigated as well. The ∙OH yield of CF NPs at 50 °C is significantly higher than that at 25 °C (Figure 3i), implying the increased temperature can promote the catalytic reaction. Similarly, when CF NPs are exposed to 808 nm light, the ability of CF to produce ∙OH is also higher than that at 25 °C, confirming the boosted effect of photothermal effect on the Fenton‐like/Fenton reaction of CF NPs. To verify this, the steady‐state catalytic kinetics experiments of CF nanoparticles at 25 and 50 °C were conducted by changing H_2_O_2_ concentrations at fixed CF nanoparticles and TMB concentrations, followed by measuring the generated hydroxyl radicals. The Michaelis–Menten constant (K*_m_*) of CF nanoparticles was calculated to be 6.53 and 5.21 mm at 25 and 50 °C by fitting the kinetic data with the Michaelis–Menten equation (Figure S12a, Supporting Information) and Lineweaver double‐reciprocal plot (Figure S12b, Supporting Information), demonstrating the improved catalytic activity of CF nanoparticles at higher temperatures. All these results verify that the as‐prepared CF could be used as a GSH‐triggered Fenton‐like/Fenton reagent for amplified CDT through GSH consumption and photothermal effect.

### 2.4 In Vitro Anticancer Effect of CFMB and Related Mechanism Exploration

**Figure 4:**
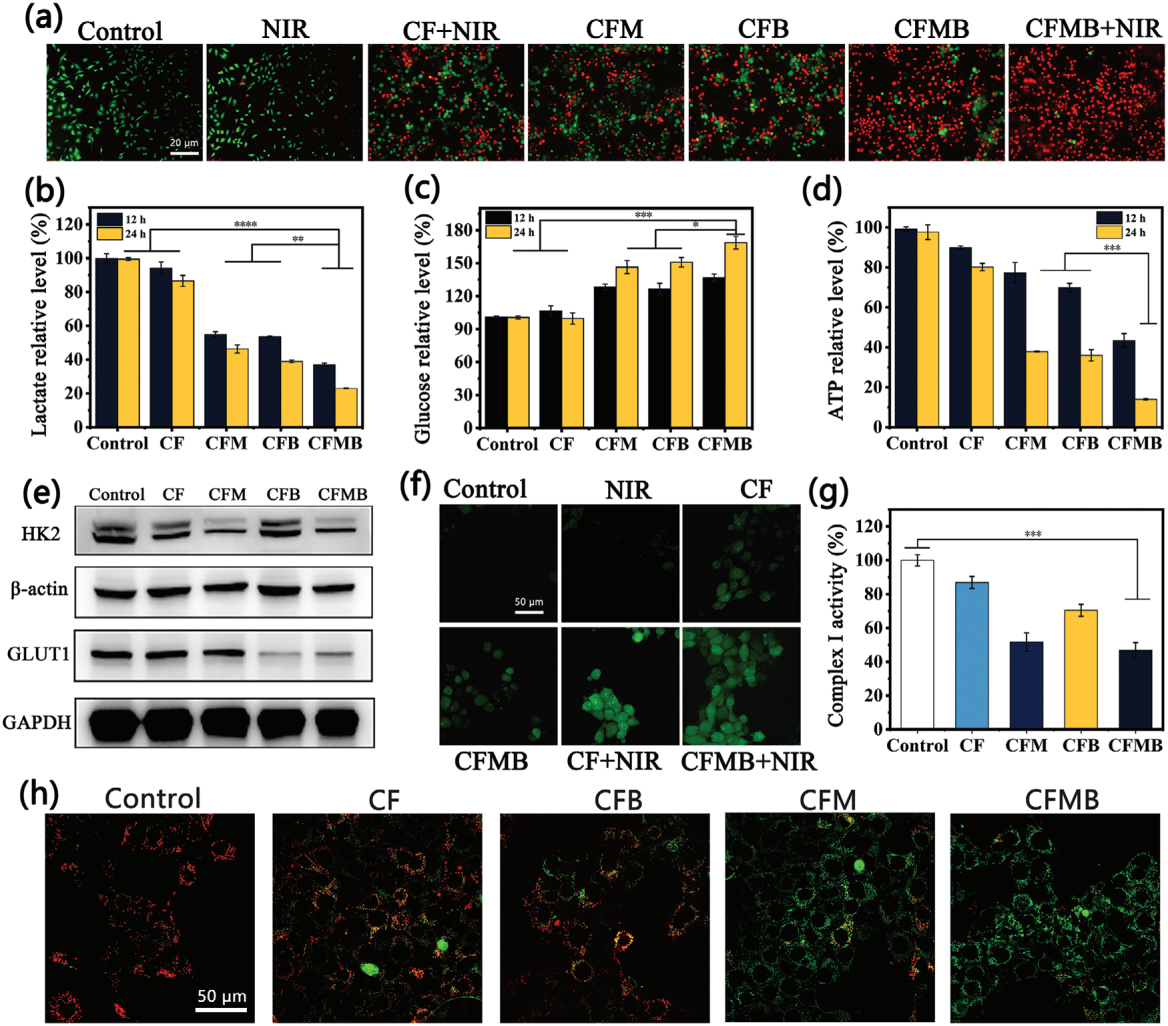
a) Fluorescence microscope images of AM/PI stained 4T1 cells after various treatments. Extracellular lactate b), glucose c), and intracellular ATP d) content of 4T1 cells subjected to diverse treatments for 12 and 24 h. e) Western blot analysis on the expressions of GLUT1 and HK2 in 4T1 cells. f) CLSM images of ∙OH detected by DCFH‐DA in 4T1 cells subjected to diverse treatments. g) Relative catalytic activity of complex I in 4T1 cells after different treatments. h) CLSM images of JC‐1 stained 4T1 cells subjected to diverse treatments.

The internalization of nanodrugs in cells is a prerequisite for therapeutic drugs to exert therapeutic effects. Therefore, cellular uptake of CFMB was first assessed by confocal laser scanning microscopy (CLSM). To be specific, 4T1 cancer cells were co‐cultured with fluorescein isothiocyanate (FITC) labeled CF (denoted as FITC‐CF) NPs and then observed using CLSM. As seen from Figure S13a (Supporting Information), obvious green fluorescence is emitted from 4T1 cells, and the fluorescence reaches the strongest after the NPs were cultured with cells for 6 h, suggesting FITC‐CF NPs could be effectively endocytosed by 4T1 cells. The results of the gray value analysis further confirm that the amount of FITC‐CF NP endocytosis is time‐dependent before 6 h (Figure S13b, Supporting Information). The biocompatibility of NPs was also evaluated before in vitro anti‐tumor experiments. HUVEC cells were selected in normal cells mode and cultured with various NPs (CF, CFM, CFB, CFMB) at the concentration of 200 µg mL^−1^. CCK‐8 assays reveal the survival rate of HUVEC cells was no ˂85% after incubation for 24 h (Figure S14, Supporting Information), indicating that all the NPs have relatively low cell toxicity. Next, the therapeutic effect of CFMB in vitro was assessed. After various treatments, 4T1 cancer cells were stained using calcein‐AM and propidium iodide (AM/PI) to mark the dead and living cells respectively, and then cell survival was observed with CLSM. As exhibited in **Figure** **4**a, the NIR group exhibits hardly cell damage, indicating NIR light alone does not cause cytotoxicity. A degree of cell death is found in the CF + NIR group because of synergistic killing effects caused by CF itself as CDT and PTT reagent. Notably, as compared with the group of CFM and CFB, the CFMB group shows a larger proportion of dead cells, indicating that the combined MET and BAY synergistic intervention of energy metabolism has a better killing effect on cancer cells. Interestingly, it is found that almost all cells in the CFMB + NIR group die, confirming that the photothermal effect can remarkably potentiate bioenergetic therapy and CDT. In addition, the CCK‐8 assay showed similar antitumor results as the AM/PI double staining assay (Figure S15, Supporting Information). All the above data indicates the remarkable therapeutic efficiency of CFMB against tumor cells.

As shown in Scheme 1, GSH‐triggered CFMB degradation releases Cu^+^/Fe^2+^ irons, MET, and BAY. Cu^+^/Fe^2+^ ions‐mediated ∙OH generation impairs OXPHOS to decrease ATP generation. MET synchronously suppresses glycolysis and OXPHOS by inhibiting HK2 activity and damaging mitochondrial function to decrease the production of ATP and lactate. In addition, BAY blocks glucose transport into tumor cells by inhibiting GLUT1 to further potentiate glycolysis repression. Based on the above mechanism, the metabolism regulation of CFMB was first investigated by assessing the variation in lactate, glucose, and ATP at the cellular level. As exhibited in Figure 4b, in contrast to the control group, the extracellular lactic acid levels in the CFM and CFB groups decline significantly, indicating effective inhibition of glycolysis. The lowest lactate level is observed in the CFMB group, which is 37% and 23% at 12 and 24 h respectively, demonstrating the synergistic effect of MET and BAY on glycolytic inhibition. Similarly, the highest extracellular glucose level is detected in CFMB‐treated cells (Figure 4c). Intracellular ATP level was also determined after distinct treatments. As displayed in Figure 4d, the CF induces a slight ATP decrease, while the CFM or CFB group has a larger decline in ATP, suggesting that impairing OXPHOS by ∙OH, inhibiting glycolysis and OXPHOS by MET and ∙OH, or blocking glucose uptake alone would aggravate energy deprivation. Notably, the ATP level of the cells treated with CFMB for 24 h decreased significantly, and only 14.1% of the ATP was produced, confirming that metabolic intervention by combining ∙OH, MET, and BAY has a synergistic effect on depriving tumor energy. To explore the molecular mechanism of CFMB NPs in metabolic intervention, western blotting analysis was conducted to evaluate the expression of proteins associated with glycolysis in 4T1 cells. Figure 4e proves that HK2 expression in CFM and CFMB groups is greatly lower than that in control and CFB groups, indicating MET could effectively induce the down‐regulation of HK2 and thus reduce the glycolytic activity. Besides, GLUT1 expression in the CFB and CFMB groups remarkably descends in comparison with that in the control group, evidencing restraining effects of BAY to GLUT1. The expressions of GLUT1 and HK2 in 4T1 cells were further quantitatively evaluated by the related gray values (Figure S16, Supporting Information), and the results were basically consistent with those observed above. These results suggest that CFMB can reduce glycolytic activity by inhibiting the expression of HK2 and GLUT1.

As depicted in Scheme 1, CFMB reacts with GSH to release Fenton‐like/Fenton reagent Cu^+^/Fe^2+^ ions that catalyze tumor‐overexpressed H_2_O_2_ to ∙OH. Therefore, 2,7‐dichlorofluorescein diacetate (DCFH‐DA) chemical probe was then used to detect the production of intracellular ∙OH, which can emit green fluorescence after ∙OH oxidation. The change of green fluorescence signal in NIR light irradiated cells is negligible (Figure 4f), indicating NIR light alone does not affect ∙OH production. Cells treated with CF or CFMB show stronger green fluorescence, which is associated with ∙OH generated by Cu^+^/Fe^2+^ mediated Fenton‐like/Fenton reaction. Notably, the strongest green fluorescence appears in the CF + NIR or CFMB + NIR light‐treated 4T1 cells, which is attributed to enhanced ∙OH production due to the combined effect of PTT, proving the increased temperature is conducive to H_2_O_2_ being catalyzed by Cu^+^/Fe^2+^ to ∙OH.

It has been reported that ROS and MET can inactivate OXPHOS‐related enzymes such as complex I blocking mitochondrial energy supply.^[^ ^28^ ^]^ Thus, we investigated whether CFMB‐mediated ∙OH production and MET release affect the activity of complex I and impaired OXPHOS. As shown in Figure 4g, CF NPs resulted in an 11.6% decline in complex I activity compared to the control group (≈100), while CFM and CFMB NPs showed significant complex I inactivation with activity values of 50.1% and 47.8%, respectively, demonstrating the generated ∙OH and released MET can impede OXPHOS. Additionally, the changes in mitochondrial membrane potential (MMP) were further detected by a JC‐1 probe to verify the mitochondrial damage. JC‐1 can form a J‐aggregate that emits red fluorescence at high MMP while remaining a monomer that emits green fluorescence at low MMP.^[^ ^29^ ^]^ As shown in Figure 4h, moderate green fluorescence is observed in the CF or CFB group, demonstrating Cu^+^/Fe^2+^ mediated ∙OH production could induce mitochondria damage. The stronger green fluorescence is observed for the CMB or CFMB group, demonstrating the synergistic effect of Cu^+^/Fe^2+^ mediated ∙OH production and MET on MMP dissipation. The results strongly imply that CFMB can effectively induce mitochondrial damage, which is promising for energy deprivation by the OXPHOS pathway.

**Figure 5:**
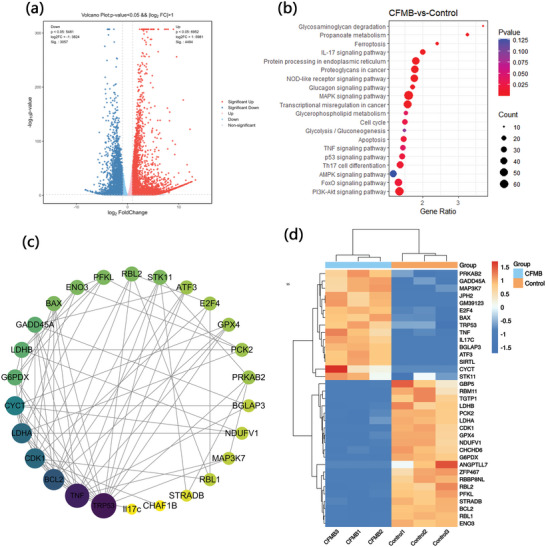
a) Volcano plot of DEGs for the control and CFMB group. b) KEGG pathway enrichment analysis after treatments. c) PPI network including these representative DEGs. d) The DEGs of interest between different groups were displayed by a heat map.

To further explore the therapeutic mechanism of CFMB, genome‐wide RNA‐sequencing was performed on the 4T1 cells treated with CFMB. As displayed in **Figure** **5**a, volcano plots present that 7551 differentially expressed genes (DEGs) are detected after treatment with CFMB, with 4494 up‐regulated genes (red dots) and 3057 down‐regulated genes (blue dots). DEGs enrichment results imply that multiple signaling pathways associated with the proliferation and death of 4T1 cancer cells are determined to be significantly changed after treatment of CFMB NPs, such as the apoptosis signaling pathway, the TNF signaling pathway, the p53 signaling pathway, the MAPK signaling pathway, the MTOR signaling pathway, cell cycle signaling pathway and so on (Figure 5b). These results indicate that these biological events may be attributable to effective anticancer treatments resulting from CFMB. It is worth noting that multiple tumor metabolism‐related gene signaling pathways are affected including the PI3K‐Akt signaling pathway, the AMPK signaling pathway glycolysis/gluconeogenesis signaling pathway, and so on. For example, the PI3K‐Akt signaling pathway plays key roles in the uptake and utilization of various nutrients involving glutamine, glucose, and nucleotides.^[^ ^30^ ^]^ Specifically, a number of DEGs significantly affect these pathways (Figure 5c,d). For example, down‐regulation of LDHA, LDHB, and PFKL genes appears to result in the inhibition of glycolysis,^[^ ^31^ ^]^ the overexpression of positively related genes STK11 and MAP3K7 accounts for the activated AMPK signaling pathway.^[^ ^32^ ^]^ In addition, DEGs involved in mitochondrial damage are regulated in the CFMB‐treated cells. Both CHCHD6 and Ndufv1, which are associated with the integrity of the mitochondrial ridge structure and the mitochondrial complex I,^[^ ^33^ ^]^ are significantly down‐regulated, resulting in affected mitochondrial function and reduced ATP production. The data demonstrates that CFMB can simultaneously restrain glycolysis and damage mitochondrial function, thus depriving the energy supply of the tumor. Furthermore, the ferroptosis signaling pathway and down‐regulated GPX4 suggest that CFMB has anticancer effects on 4T1 cancer cells through GSH depletion and ROS elevation.^[^ ^34^ ^]^ Overall, all these results further reveal the anti‐tumor mechanism of CFMB is related to glycolysis and mitochondrial damage‐mediated energy metabolic intervention and GSH‐enhanced CDT.

All the above data verify the therapeutic mechanism illustrated in Scheme 1. Tumor‐overexpressed GSH triggers CFMB degradation to release Cu^+^/Fe^2+^ irons, MET, and BAY. Cu^+^/Fe^2+^ ions‐mediated ∙OH generation impairs OXPHOS by reducing complex I activity. The MET simultaneously restrains glycolysis and OXPHOS by inhibiting HK2 and damaging mitochondrial function, respectively. BAY blocks glucose uptake by inhibiting GLUT1 expression to further potentiate the glycolysis repression. Therefore, synchronous interventions of glycolysis (targets of HK2 and GLUT1) and OXPHOS (inhibitors of ∙OH and MET) through dual pathways were achieved to boost energy deprivation of tumor cells. Moreover, the inherent physicochemical properties of CF NPs enable amplified CDT with concurrent GSH depletion and photothermal effects. As a result, CFMB achieves potent antitumor efficacies by boosting bioenergetic therapy and CDT/PTT in a mutually reinforcing way.

### 2.5 In Vivo Antitumor Effects of CFMB NPs

**Figure 6:**
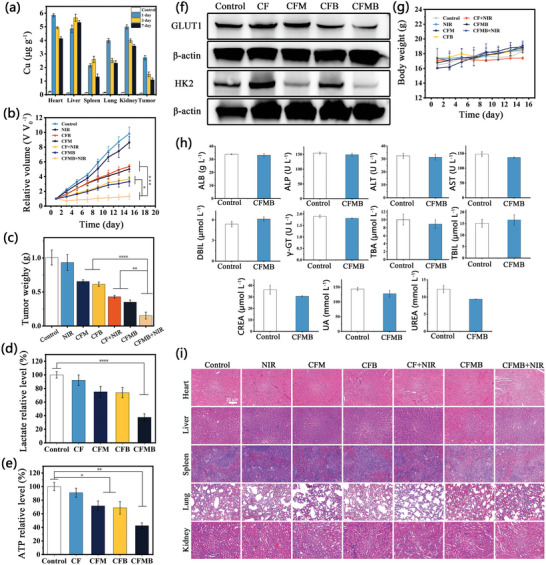
a) Biodistribution of Cu in mice at day 1, 3, and 7 days after administration. b) The relative 4T1 tumor volume after different treatments. c) The average tumor weight in various groups. The relative d) lactate and e) ATP levels in the harvested tumors after different treatments. f) Western blot analysis on the expressions of GLUT1 and HK2 in the harvested tumors. g) Body weight variation of mice from different groups. h) Hematological parameters of mice after being treated with CFMB plus NIR light. i) H&E staining images of major organs after various treatments.

The prerequisite for the therapeutic effects of nanodrugs is their effective accumulation at the tumor site. Therefore, we first evaluated the biodistribution of CFMB NPs in tumor‐bearing mice before in vivo therapy experiments. The CFMB was administered to mice via the tail vein and then mice were sacrificed Cu level in major organs and tumor tissue was detected by ICP‐MS at scheduled time. As exhibited in **Figure** **6**a, the CFMB distribution in tumors reaches its maximum 24 h after administration, demonstrating that CFMB might assemble in tumor tissue via enhanced permeability and retention effect (EPR) due to the applicable size. Meanwhile, it is worth noting that CFMB distribution in major organs shows a first increasing and then declining trend with time extension. As time extends to 7 days, CFMB NPs could be excreted from mice, manifesting the clearance process is time‐dependent, which is similar to the scavenging behavior of some reported nano‐drugs.

Based on the efficient in vitro therapeutic capability and tumor accumulation of CFMB NPs, the in vivo antitumor effect of CFMB was further assessed in a 4T1 tumor xenograft mouse model. All tumor‐bearing mice were divided into 7 groups: PBS, NIR, CF + NIR, CFM, CFB, CFMB, and CFMB + NIR. During the whole treatment course, the corresponding nano‐drug was administered to mice via the tail vein once (10 mg kg^−1^). For mice requiring laser treatment, the tumor sites were irradiated once 24 h after administration. Their body weights as well as tumor volumes were monitored every other day during treatment. Figure 6b proves that NIR light irradiation alone has no antitumor effect. CFMB NPs exhibit a better tumor suppression effect than either CFM or CFB NPs due to the much more tumor energy deprivation caused by the synergistic metabolic intervention of MET and BAY. Besides, CF + NIR light irradiation shows an almost identical inhibitory effect on tumor growth as CFMB, which is related to PTT and Cu^+^/Fe^2+^ mediated CDT. Notably, CFMB + NIR light exhibits the best effect on the suppression of tumor growth, revealing the positive role of PTT in synergistic bioenergetics therapy and CDT. At the end of treatment, the digital photography of the dissected representative tumor in each group basically corresponds with the result of tumor growth inhibition (Figure S17, Supporting Information). The antitumor effect is further validated by measuring the average weight of mouse tumors dissected after treatment. The average tumor weight of CFMB + NIR light‐treated mice is the lowest (Figure 6c), agreeing with tumor growth suppression. In order to further explore the relevant mechanisms of CFMB NPs in metabolic intervention, the metabolic changes of tumors in vivo were investigated. As displayed in Figure 6d and Figure 6e, the lactic acid and ATP levels in the tumors dissected from mice treated with CFMB NPs were significantly decreased, which is basically consistent with the results of in vitro experiments. Western blotting analysis of HK2 and GLUT1 of dissected tumors was conducted (Figure 6f). Compared with the control group, both HK2 and GLUT1 expressions were down‐regulated in the CFMB group, demonstrating the inhibitory effect of CFMB on glycolysis.

The in vivo biocompatibility of CFMB NPs was systematically assessed. Firstly, during the whole treatment period, the mice in all experimental groups maintained a slight trend of weight gain, and no weight loss was observed, implying the adopted therapy did not induce serious systemic toxicity (Figure 6g). In addition, the blood samples of the mice 14 days after intravenous injection were analyzed. As shown in Figure 6h, the eight indexes representing liver function and three indexes representing renal function have no abnormal difference from the control group, further proving the high hemocompatibility, ignorable nephrotoxicity, and low hepatotoxicity of CFMB NPs. Furthermore, hematoxylin and eosin (H&E) staining of major organs including heart, liver, lung, spleen, and kidney presents negligible histological changes and normal cellular morphology (Figure 6i). All the above results demonstrate that the designed CFMB nano‐drug possesses decent in vivo biocompatibility and biosafety.

## 3 Conclusion

In summary, we developed a CFMB nanoplatform to boost energy deprivation by synchronous interventions of glycolysis and OXPHOS for antitumor bioenergetic therapy synergetic with CDT/PTT. CFMB can efficiently deliver drugs to the tumor area, where it is subsequently triggered by tumor‐overexpressed antioxidant GSH to degrade and release MET and BAY. The released MET simultaneously restrains glycolysis and OXPHOS by inhibiting HK2 activity and damaging mitochondrial function to deprive energy, respectively. Meanwhile, BAY blocks glucose uptake by inhibiting GLUT1, further potentiating the glycolysis repression. Moreover, GSH‐triggered CFMB degradation releases Cu^+^/Fe^2+^ ions to catalyze tumor‐overexpressed H_2_O_2_ to ∙OH, which not only achieves GSH‐depletion amplified CDT but also impairs OXPHOS. As a result, the multiple pathways of metabolism interference significantly boosts energy deprivation of tumorigenic energy sources. Furthermore, the photothermal effect of CFMB could both directly kill cancer cells and promote ∙OH generation. In vitro and in vivo experiments concurrently manifest that CFMB achieves potent antitumor efficacies against 4T1 breast tumors without causing systemic toxicity. Therefore, this study might propose a valid strategy for boosting bioenergetic‐based cancer therapy via integrating multiple pathways of metabolic intervention and PTT/CDT therapy.

## 4 Experimental Section

### Materials

H_2_O_2_ (30%), ethanol, metformin hydrochloride (MET), 5,5′‐Dithiobis‐(2‐nitrobenzoicacid) (DTNB), Bay‐876 (BAY), 3,3,5,5‐tetramethylbenzidine (TMB) and cupric chloride dihydrate (CuCl_2_∙2H_2_O) were ordered from Aladdin Reagent Co., Ltd. (China). Calcein acetoxymethyl ester (calcein‐AM), 2,7‐Dichlorofluorescein diacetate (DCFH‐DA), glutathione (GSH), propidium iodide (PI) and 4′,6‐diamidino‐2‐phenylindole (DAPI) were ordered from Sigma‐Aldrich. (St. Louis, MO). Iron chloride hexahydrate (FeCl_3_∙6H_2_O), urea (CH_4_N_2_O), and polyacrylamide ([CH_2_CH(CONH_2_)]n) were supplied by Macklin Biochemical Technology Co., Ltd. (China)., ATP assay kit and glucose assay kit were purchased from Beyotime Biotech. Inc. (China). Lactate assay kit bought from Nanjing Jiancheng Bioengineering Institute (China). GLUT1/SLC2A1 Rabbit pAb, Hexokinase II Rabbit pAb, and GAPDH Rabbit pAb were purchased from Abclonal Technology.

### Characterizations

The zeta potential and size of NPs were tested via dynamic light scattering (DLS, NanoZS90, Malvern). Their morphologies were characterized by transmission electron microscopy (TEM, Tecnai F20, US) and scanning electron microscopy (SEM, US‐70, Hitachi). Their crystal was recorded via an X‐ray diffractometer (XRD, BRUKERD8 Advance). The UV–vis–NIR absorption spectrum of NPs was recorded by UV–vis–NIR spectrophotometer (UV–vis, UV‐1300, Shimadzu). The valence state of the element was analyzed by X‐ray Photoelectron Spectroscopy (XPS, AXIS Ultra DLD, Shimadzu).

### Preparation of CF NPs

The CF NPs were synthesized using the hydrothermal method.^[^ ^35^ ^]^ Firstly, CuCl_2_·2H_2_O (0.25 mmol) and FeCl_3_·6H_2_O (0.83 mmol) were stirred in deionized water (15 mL). Subsequently, trisodium citrate dihydrate (1.5 mmol), urea (2.25 mmol), and polyacrylamide (225 mg) were successively added to the above solution for further 1 h stirring. After a 12 h hydrothermal reaction at 473 K, the obtained CF was magnetically separated and washed using ethanol three times.

### Preparation of CFMB NPs

MET (3 mg) and BAY (1 mg) were successively added to the methanol dispersion of CF NPs (20 mg) for 24 h stirring at 25 °C. Next, prepared CFMB NPs were isolated via magnetism with distilled water washing 3 times, and then freeze‐dried. According to the difference between the supernatant collected after centrifugation and the initial solution at 290 nm, the loading rate (LC) of BAY was determined by the standard calibration curve. The LC of MET was determined as follows: CFMB NPs were dissolved in GSH (1 mm) to induce the degradation of CFMB and the complete release of MET. After the determination of their 230 nm absorption value, the LC of MET was obtained according to MET standard curves.

### MET and BAY Release from CFMB NPs

Phosphate buffer solution (PBS) was used to simulate a complex physiological environment. 10 mg of CFMB NPs were dispersed into 2 mL of PBS with a simulated TME condition (pH 6.5, GSH 10 mm) and kept continuous shaking. At the predetermined time point, 100 µL of solution was taken out for detection and filled with the same volume of fresh PBS. The cumulative quantity of MET and BAY released from NPs was calculated according to the UV–vis absorption intensity of MET and BAY at 230 and 290 nm, respectively.

### Photothermal Performance Evaluation

CFMB NP aqueous solution with various concentrations was illuminated by 808 nm light, and its temperature variation was detected by a temperature sensor. Additionally, the photothermal stability is assessed by 808 nm light on/off cycle. Moreover, CFMB NPs were 808 nm light irradiated and then cooled down at room temperature to estimate the photothermal conversion efficiency based on the reported method.^[^ ^36^ ^]^

### ∙OH Detection

A total of 2 mL of CF (50 µg mL^−1^) aqueous solution containing GSH (10 mm) or (0 mm) were shaken for 10 min, and then TMB (4.17 mm) and H_2_O_2_ (1 mm) were added, followed by testing the absorbance at 652 nm. To research GSH influences on ∙OH generation, 652 nm adsorption variations of TMB in 2 mL of CF (50 µg mL^−1^) aqueous solution containing TMB (4.17 mm), H_2_O_2_ (1 mm) and various concentrations of GSH (1, 2, 4, 8, 10 and 15 mm) were tested. For estimating the role of temperature on ∙OH generation, the CF dispersion containing GSH, H_2_O_2,_ and TMB was put in a 50 °C water bath or exposed to 808 nm light and then centrifuged to test the UV–vis absorption of the supernatant.

### In Vitro Therapy Effect

Live/dead cell staining experiments were carried out to evaluate the in vitro therapy effect. To be specific, 4T1 cells were put in 96‐well culture plates to grow overnight. Next, various treatments including NIR light, CF + NIR light, CFM, CFB, CFMB, or CFMB + NIR light respectively were applied. For experiments requiring NIR light, the 1.25 W cm^−2^ 808 nm laser would be used to illuminate cells within 10 min. After 24 h of treatment, calcein‐AM (2 µm) and PI (6 µm) were added to dye cells. The survival and death of cells were observed by an inverted fluorescence microscope.

### Detection of Glucose, Lactic Acid, and ATP

4T1 cancer cells were added to 6‐well plates and cultured overnight. Then various treatments including CF, CFM, CFM, or CFMB for 12 or 24 h respectively were applied. Following that, the cell culture medium was measured by the Lactate Assay Kit and Glucose Assay Kit following the instructions, respectively. For ATP, after different treatments, cells were washed using PBS and collected through trypsin digestion. The intracellular ATP content of different groups was determined by the ATP Assay Kit.

### Western Blot Analysis

The 4T1 cells were added to 6‐well plates and subjected to various treatments including CF, CFM, CFM, or CFMB for 12 or 24 h, respectively. Next, cells are digested and collected to extract proteins. A BCA protein assay kit has been employed in measuring the extracted protein concentrations. Subsequently, 15 µg isolated protein was blocked into the PVDF film. After that, PVDF film was cultured with the corresponding primary antibody and HRP‐conjugated secondary antibody. Last, an improved chemiluminescence system has been employed to visualize protein bands. The protein expressions were evaluated by analyzing the gray level.

### Detection of Intracellular ∙OH

The 4T1 cells were added to 6‐well culture plates to grow overnight. Next, NIR light, CF, CFMB, CF + NIR light, as well as CFMB + NIR light for 6 h respectively were applied. Finally, cells were stained by DCFH‐DA (5 µm) for 30 min and imaged with CLSM.

### In Vivo Antitumor Study

Balb/c mice have been ordered by Zhejiang Wei Tong Li Hua Biotechnology Co., Ltd. Animal‐related experiments have been conducted according to the protocols sanctified by the Animal Experiment Ethics Committee of Ningbo University. To establish a tumor model, 5 × 10^6^ of 4T1 cells were injected subcutaneously into the right hind of each mouse. As tumor volume grew about 100 mm^3^, 7 groups (5 mice for each group) were randomly divided and received diverse treatments as follows: 1) PBS, 2) PBS + NIR light, 3) CF + NIR light, 4) CFM, 5) CFB, 6) CFMB, and 7) CFMB + NIR light. The corresponding nanodrug in 0.1 mL of PBS was injected into each mouse through the tail vein. The time lag between material injection and the initiation of NIR light (0.5 W cm^−2^) in animal experiments is 24 h. Their body weights as well as tumor volumes were monitored every other day during treatments.

### RNA‐Sequencing Analysis

The 4T1 cancer cells were added to 6‐well plates and cultured overnight. CFMB NPs were next added. After another 24 h treatment, total RNA was extracted for RNA‐sequencing analysis (OEbiotech, China). An R package, DESeq, was conducted to determine the differentially expressed genes (DEGs), using a cut‐off of P < 0.05. The heatmaps were built with R programming. The DEGs with | log2 (foldchange) | > 1 and P < 0.05 were used for Gene Set Enrichment Analysis (GSEA) via the Pathway enrichment analysis package in R software.

### Statistical Analysis

All data results were exhibited as the mean ± standard deviation by three parallel experiments. The statistical comparisons between experimental groups were determined by the Student's t‐tests. ^*^ *p* < 0.05, ^**^ *p* < 0.01, and ^***^ *p* < 0.001 were supposed to significant differences.

## Conflict of Interest

The authors declare no conflict of interest.

## Supporting information

Supporting Information

## Data Availability

The data that support the findings of this study are available from the corresponding author upon reasonable request.
