# Supplementary material for: Boosting Energy Deprivation by Synchronous Interventions of Glycolysis and Oxidative Phosphorylation for Bioenergetic Therapy Synergetic with Chemodynamic/Photothermal Therapy
Source: Adv Sci (Weinh). 2024 Mar 15;11(23):2401738. doi: 10.1002/advs.202401738 (PMC11187878; doi:10.1002/advs.202401738)
Supplement: Supplementary file 1 — Supporting Information [file ADVS-11-2401738-s001.pdf]

ADVANCED SCIENCE Open Access

Supporting Information

for *Adv. Sci.*, DOI 10.1002/advs.202401738

Boosting Energy Deprivation by Synchronous Interventions of Glycolysis and Oxidative Phosphorylation for Bioenergetic Therapy Synergetic with Chemodynamic/Photothermal Therapy

Xiangjun Wei, Renlu Han, Yun Gao, Pengxin Song, Zhen Guo, Yafei Hou*, Jiancheng Yu and Keqi Tang**

Supporting Information

Boosting energy deprivation by synchronous interventions of glycolysis and oxidative phosphorylation for bioenergetic therapy synergetic with chemodynamic/photothermal therapy

Xiangjun Wei, Renlu Han, Yun Gao, Pengxin Song, Zhen Guo, Yafei Hou,* Jiancheng Yu, Keqi Tang**

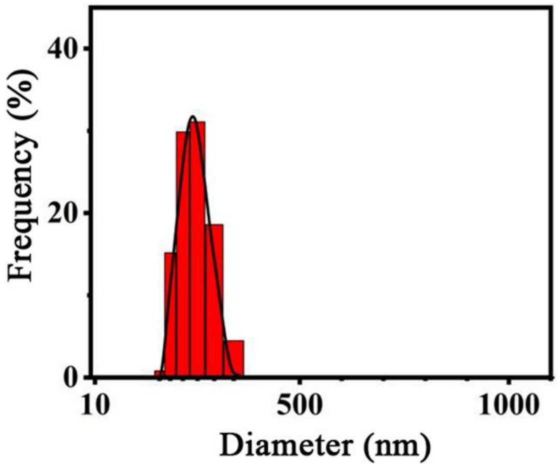

Figure S1. The size distribution of CF NPs based on SEM image.

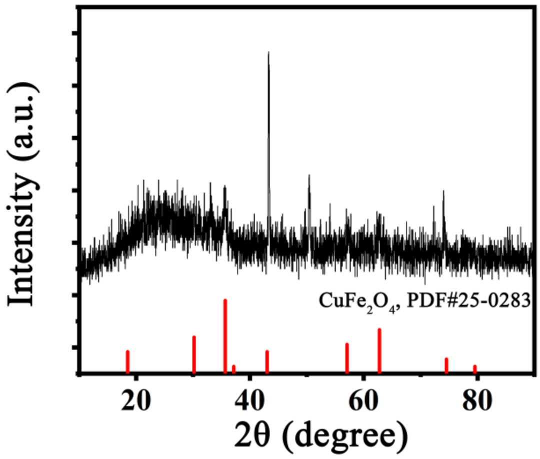

Figure S2. XRD spectra of CF NPs.

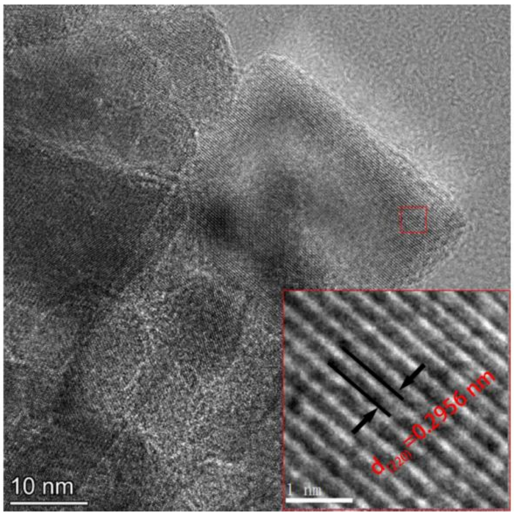

Figure S3. High-angle annular dark field TEM image of CF NPs.

Element	Wt%	At%
O	40.93	55.94
Fe	34.97	27.44
Cu	24.10	16.62
Matrix	Correction	ZAF

Figure S4. Element compositions of Fe, Cu and O in CF NPs.

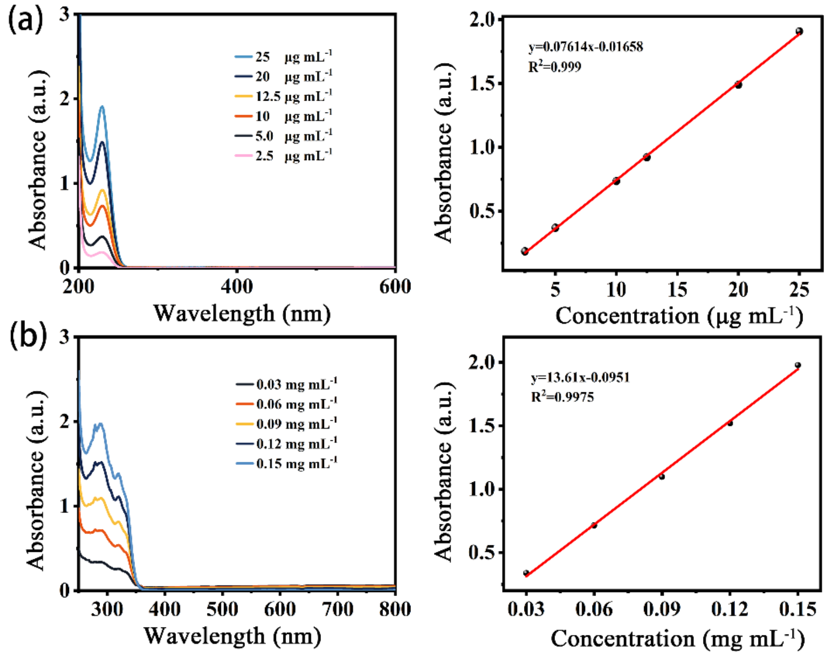

Figure S5. (a) The UV-vis spectra of MET at various concentrations and MET-concentration dependent calibration curve of the absorbance at 230 nm. (b) The UV-vis spectra of BAY at various concentrations and BAY-concentration dependent calibration curve of the absorbance at 290 nm.

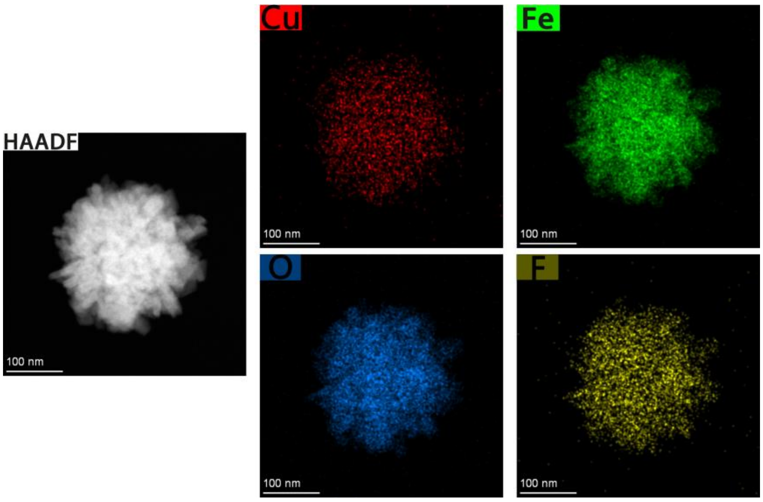

Figure S6. High-angle annular dark field (HAADF) TEM image and elemental mapping of CF NPs.

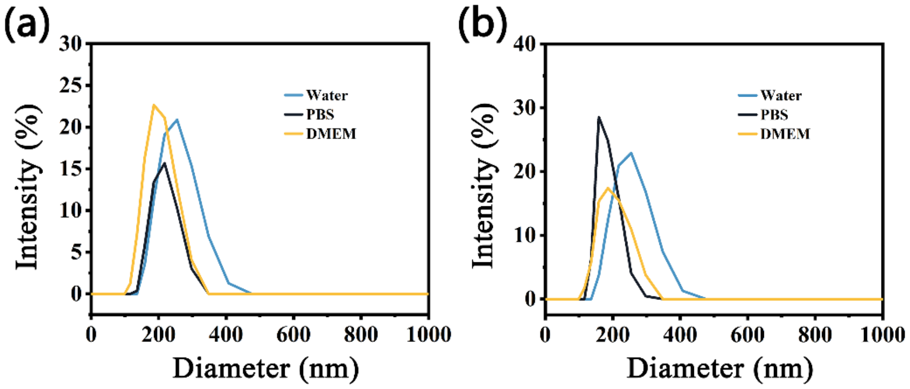

Figure S7. The hydrodynamic diameter of CF NPs in Water, PBS and DMEM. (a) The first day and (b) the seventh day.

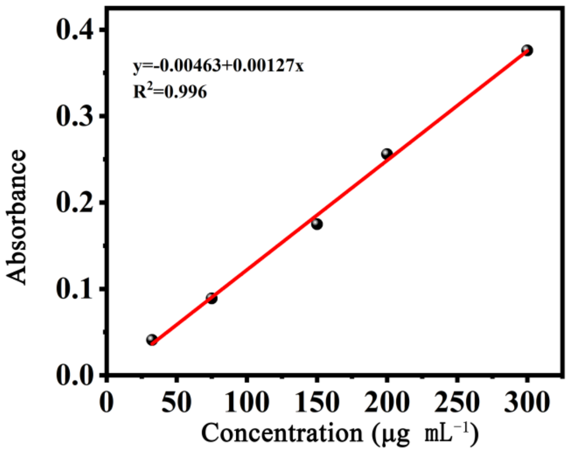

Figure S8. Linear fitting curve of absorbance value at 808 nm for CF NPs with different concentrations.

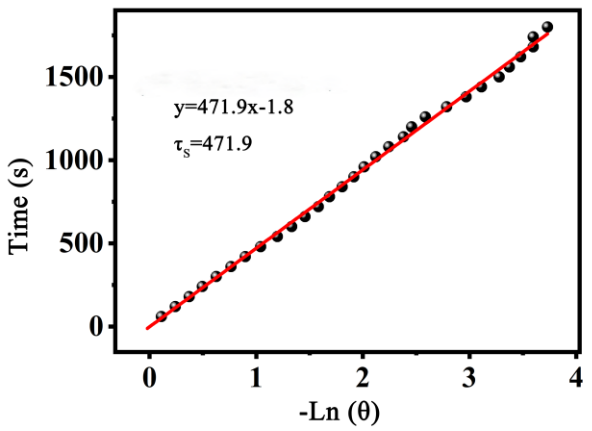

Figure S9. Time constant (s) for heat transfer from the system was determined to be τ_s = 471.9 s by applying the linear time data from the cooling period, which was obtained from the cooling stage.

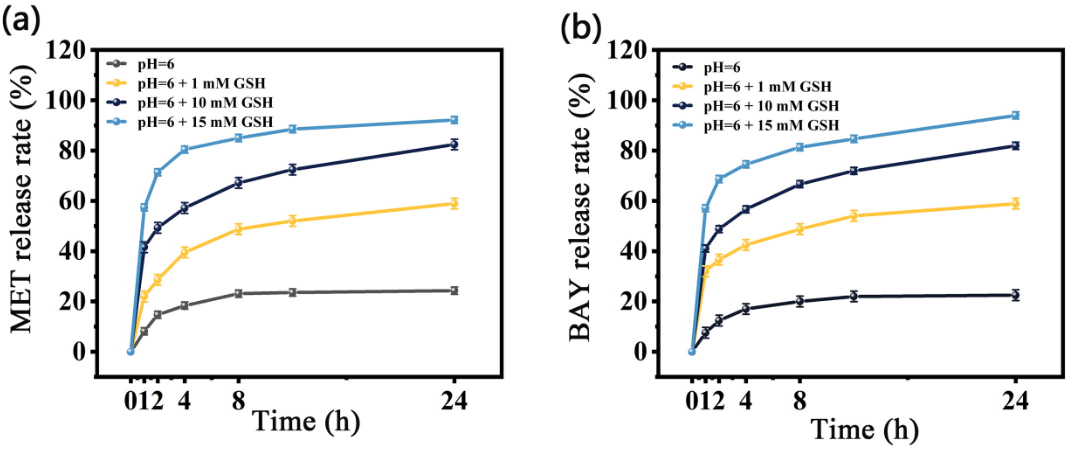

Figure S10. The release profile of (a) MET and (b) BAY at different GSH concentrations.

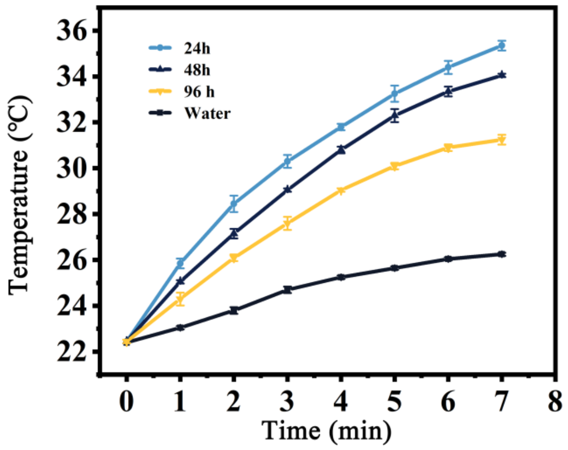

Figure S11. The photothermal curves of the CF NPs after reacting with GSH for different time (24, 48, 96 h).

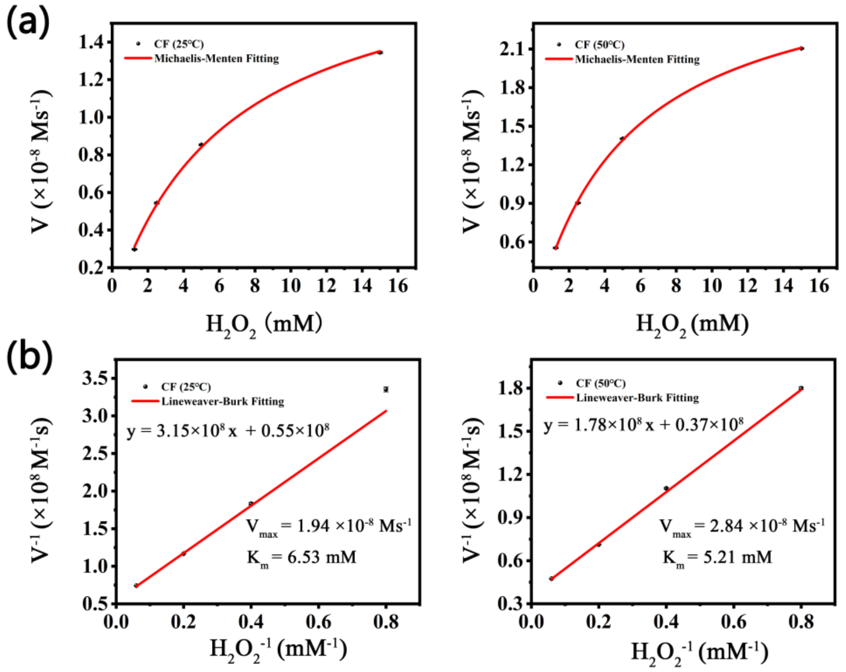

Figure S12. (a) Michaelis-Menten kinetic analysis and (b) Lineweaver-Burk plotting of catalytic activity of CF NPs at 25 °C and 50 °C.

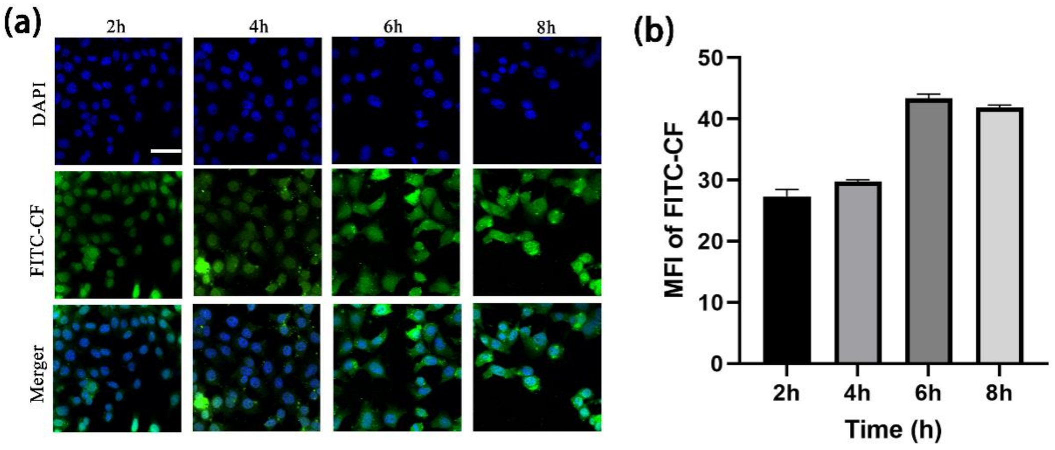

Figure S13. (a) CLSM images of 4T1 cells treated with FITC-CF NPs for 2, 4, 6, and 8 h. (Scale bar = 50 um). (b) The gray values corresponding to CLSM images of 4T1 cells treated with FITC-CF NPs for different time.

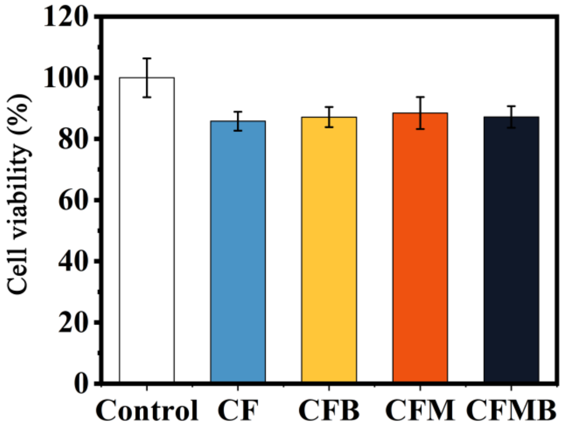

Figure S14. Cell viability of HUVEC cells treated with various NPs (CF, CFM, CFB, CFMB) at the concentration of 200 μg mL^{-1} for 24 h.

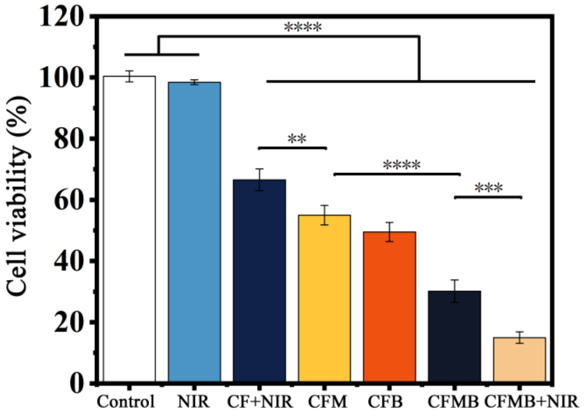

Figure S15. Cell viability of 4T1 cells treated with various NPs (CF, CFM, CFB, CFMB) at the concentration of 200 μg mL^{-1} for 24 h.

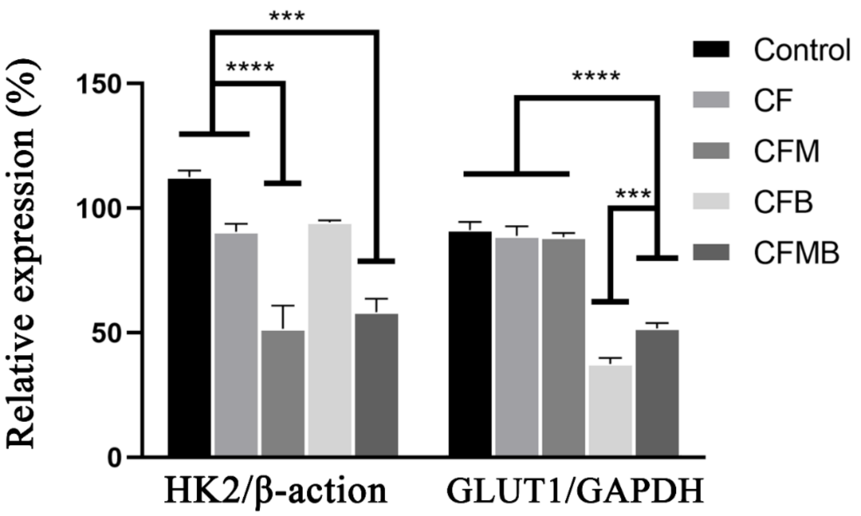

Figure S16. The relating gray values of western blot analysis on the expressions of GLUT1 and HK2 in 4T1 cells.

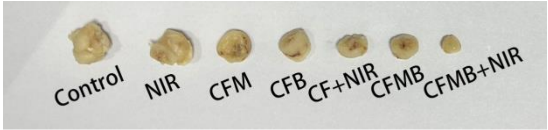

Figure S17. The digital photography of the dissected representative tumor in different groups.
